# Acidosis in the critically ill - balancing risks and benefits to optimize outcome

**DOI:** 10.1186/cc13815

**Published:** 2014-04-03

**Authors:** Gerard F Curley, John G Laffey

**Affiliations:** 1 Department of Anesthesia, Keenan Research Centre for Biomedical Science of St Michael’s Hospital, St Michael's Hospital, 30 Bond Street, Toronto M5B 1 W8, ON, Canada; 2 Department of Anesthesia, University of Toronto, Room 121, Fitzgerald Building, 150 College Street, Toronto M5S 3E2, ON, Canada

## Abstract

Acidosis is associated with poor outcome in critical illness. However, acidosis - both hypercapnic and metabolic - has direct effects that can limit tissue injury induced by many causes. There is also a clear potential for off-target harm with acute exposure (for example, raised intracranial pressure, pulmonary hypertension), and with exposure for prolonged periods (for example, increased risk of infection) or at high doses. Ongoing comprehensive determination of molecular, cellular and physiologic impact across a range of representative pathologies will allow us to understand better the risks and benefits of hypercapnia and acidosis during critical illness.

## 

The effects of acidosis in critical illness are complex and depend on multiple factors, including the etiology of the acidosis (hypercapnic versus metabolic), the degree of acidosis and whether the acidosis accumulates acutely or more gradually, where compensatory or adaptive mechanisms may blunt its effects. While acidosis is traditionally associated with poor outcome in critical illness, it is not clear to what extent - if any - this is a causal relationship. In any case, simple avoidance of acidosis is generally not feasible in the critically ill. In fact, conventional protective ventilation strategies that minimize lung stretch increase the prevalence of hypercapnic acidosis (HCA) in the critically ill, and have been clearly demonstrated to save lives in patients with acute respiratory distress syndrome. However, there is also a clear potential for harm with HCA or metabolic acidosis (MAC), whether due to acute exposure (for example, raised intracranial pressure, pulmonary hypertension), exposure for prolonged periods of time (for example, increased risk of infection) or high concentrations. These complexities are further underlined by the findings in multiple preclinical studies that acidosis may have direct effects which can protect cells and organs in the setting of acute organ injury. Thus, managing acidosis is an integral component of critical care, and efforts to deepen our understanding of its consequences are therefore welcome.

Stengl and colleagues report a study into the effects of relatively severe (pH 7.1) HCA and MAC on the cardiovascular system and distal organ perfusion in the healthy pig [1]. They found that acute HCA or MAC reduced cardiac contractility, but that cardiac output was maintained as a result of increased heart rate and, in the case of HCA, reduced systemic vascular resistance. They examined regional perfusion, and found that HCA - but not MAC - increased portal and carotid arterial flow, while neither HCA nor MAC affected renal blood flow. Finally, they examined right ventricular trabeculae from these animals to evaluate contractile force generation during normal pH and acidosis. Their study raises a number of key issues with regard to the hemodynamic effects of acidosis that deserve further consideration.

The effects of hypercapnia and acidosis on pulmonary hemodynamics are important, given the prevalence of pulmonary arterial hypertension in acute respiratory distress syndrome and other critical illnesses [2]. HCA and MAC increased pulmonary artery pressures to a comparable extent, with MAC primarily increasing pulmonary vascular resistance while HCA increased both cardiac output and pulmonary vascular resistance [1]. Elevated pulmonary vascular pressure has the potential to increase right heart strain and worsen clinical outcomes. However, the fact that HCA increased cardiac output despite raising pulmonary vascular resistance suggests that right ventricular function was preserved or even enhanced. In addition, we know from other studies that the effects of HCA on pulmonary hemodynamics may be less significant in pre-existing pulmonary hypertension [3]. Hypercapnia is a less potent pulmonary vasoconstrictor than hypoxia, and its physiologic role may be in augmenting hypoxic vasoconstriction [4], enhancing matching of pulmonary perfusion to ventilation and thereby increasing arterial oxygen partial pressure [5,6]. Finally, HCA may actually attenuate the development of hypoxia-induced pulmonary hypertension by reducing oxidant stress [7].

The contrasting effects of HCA and MAC in the systemic circulation are of interest. While both HCA and MAC have a direct negative inotropic effect, the indirect hypercapnia-mediated sympatho-adrenal effects of an increased heart rate and a decreased afterload lead to a net increase in cardiac output [1]. As the authors note, cardiac output could be impaired in patients for whom sympatho-adrenal mediated increases in heart rate are not achievable. However, it is somewhat reassuring to note that HCA can increase tissue oxygen delivery through other mechanisms. As discussed, HCA increases arterial oxygen tension by improving ventilation/perfusion ratio (V/Q) matching [4,8]. HCA also shifts the oxyhemoglobin dissociation curve rightwards (that is, the Bohr effect), thereby increasing tissue oxygen availability [9]. HCA may acutely elevate hematocrit by a mechanism that might involve sympathetically mediated auto-transfusion [10], further increasing tissue oxygen delivery. Acidosis may also reduce cellular respiration and oxygen consumption [11], which may further benefit a supply/demand imbalance. In addition, HCA increases oxygen tension in both subcutaneous tissues and in the intestinal wall [12]. Nonetheless, the findings of this study suggest that the judicious use of HCA in patients with maximally activated adrenergic systems (heart failure or sepsis) is advised.

The differential effects of HCA on systemic organ blood flow in this study (increased portal venous blood flow, unchanged renal blood flow) emphasizes the need for integrative studies of this kind to clearly outline the risks and benefits of HCA, particularly off-target effects. A structured approach should incorporate ongoing comprehensive determination of molecular, cellular and tissue impact across a range of representative pathologies.

These findings provide important additional insights into the hemodynamics of MAC and HCA. More broadly, they reinforce the concept that acidosis - whether metabolic or hypercapnic - exerts potent physiologic effects, and these effects may result in benefit or harm, particularly in the critically ill. We need to think about the use of hypercapnia just as we would any therapy. Consider in this regard the immunologic effects of HCA, whereby its potent immunosuppressive effects decrease lung injury in preclinical studies of ventilation-induced lung injury, ischemia–reperfusion injury, and early pneumonia and systemic sepsis [13,14]. However, as with any pharmacologic agent, dose and timing are important. The benefits of short-term application of HCA may be offset when the altered carbon dioxide state is allowed to persist. This may be particularly true where an intact host immune response is central to effective bacterial clearance. In this regard, the finding that HCA worsens prolonged untreated pneumonia [15] can be predicted from knowledge of its mechanisms of action. The importance of dose is evidenced by the finding that carbon dioxide partial pressure levels of 120 mmHg cause mitochondrial dysfunction [16]. Even permissive hypercapnia must have safe and rational upper limits.

In summary, acidosis remains prevalent in the critically ill, and advances in our understanding of the effects of acidosis help us to rationally balance the risks and benefits of acidosis in our patients. Our research efforts should focus on fully understanding the potential mechanisms by which hypercapnia and acidosis contribute to the pathogenesis of organ injury and disease. Such insights should advance our understanding of the situations in which hypercapnia may be helpful or dangerous, and should guide clinicians in regard to the rational management of carbon dioxide tension in diverse disease states.

### Abbreviations

HCA: Hypercapnic acidosis; MAC: Metabolic acidosis.

### Competing interests

The authors declare that they have no competing interests.
